# Mitochondria in Cell Senescence: Is Mitophagy the Weakest Link?

**DOI:** 10.1016/j.ebiom.2017.03.020

**Published:** 2017-03-14

**Authors:** Viktor I. Korolchuk, Satomi Miwa, Bernadette Carroll, Thomas von Zglinicki

**Affiliations:** The ABC – Newcastle University Ageing Biology Centre, Newcastle University Institute for Ageing, UK

**Keywords:** Senescence, Mitophagy, Mitochondria, Aging

## Abstract

Cell senescence is increasingly recognized as a major contributor to the loss of health and fitness associated with aging. Senescent cells accumulate dysfunctional mitochondria; oxidative phosphorylation efficiency is decreased and reactive oxygen species production is increased. In this review we will discuss how the turnover of mitochondria (a term referred to as mitophagy) is perturbed in senescence contributing to mitochondrial accumulation and Senescence-Associated Mitochondrial Dysfunction (SAMD). We will further explore the subsequent cellular consequences; in particular SAMD appears to be necessary for at least part of the specific Senescence-Associated Secretory Phenotype (SASP) and may be responsible for tissue-level metabolic dysfunction that is associated with aging and obesity. Understanding the complex interplay between these major senescence-associated phenotypes will help to select and improve interventions that prolong healthy life in humans.

**Search strategy and selection criteria:**

Data for this review were identified by searches of MEDLINE, PubMed, and references from relevant articles using the search terms “mitochondria AND senescence”, “(autophagy OR mitophagy) AND senescence”, “mitophagy AND aging” and related terms. Additionally, searches were performed based on investigator names. Abstracts and reports from meetings were excluded. Articles published in English between 1995 and 2017 were included. Articles were selected according to their relevance to the topic as perceived by the authors.

## Highlights

• Perturbed mitophagy contributes to mitochondrial dysfunction in cell senescence.
• Mitochondrial dysfunction drives multiple characteristic phenotypes of senescent cells.
• Senescence-associated mitochondrial dysfunction may be an important cause for metabolic compromise in aging.

## 1 Introduction

Senescent cells accumulate with age in a wide range of tissues. Frequencies in excess of 5%, and sometimes as much as 20% and more, have been reported in tissues from old animals both with high (white blood cells (Akbar et al., 2016); crypt enterocytes (Jurk et al., 2014, Wang et al., 2009)) and low (dermal fibroblasts (Dimri et al., 1995), hepatocytes (Jurk et al., 2014, Wang et al., 2009), fat progenitors (Schafer et al., 2016), osteocytes (Farr et al., 2016)) proliferation rates as well as in postmitotic tissues (neurons (Jurk et al., 2012)). The rate of accumulation of senescent cells in liver and intestinal crypts predicts median and maximum lifespan of mice in cohorts with widely different aging rates (e.g. late generation *TERC −/−* vs wt and dietary restricted C57Bl/6) (Jurk et al., 2014). More importantly, interventions that selectively ablate senescent cells by genetic and/or pharmacologic means may improve healthspan and lifespan in mice (Baker et al., 2016, Demaria et al., 2017, Xu et al., 2015).

Mechanistically, the age-promoting effects of senescence are associated with the restriction of regenerative capacity of stem and progenitor cells (Choudhury et al., 2007, Jurk et al., 2014) as well as the secretion of bioactive molecules (the so-called SASP (Coppe et al., 2008)), specifically pro-inflammatory and matrix-modifying peptides. Pro-aging effects of senescent cells are aggravated by SASP and, possibly, other paracrine mediators which can propagate senescence from cell to cell as a bystander effect (Nelson et al., 2012). In recent years, evidence has been mounting that senescent cells impact on their environment via yet another principal pathway: mitochondrial dysfunction.

Along with cell senescence, mitochondrial dysfunction is another essential ‘hallmark of aging’ (Lopez-Otin et al., 2013), and the two have been independently identified as important drivers of aging (Finkel, 2015). Importantly, they are closely interlinked: mitochondrial dysfunction drives and maintains cell senescence (Correia-Melo et al., 2016, Passos et al., 2007, Wiley et al., 2016), while at the same time cell senescence, specifically persistent DNA damage response signalling, directly contributes to Senescence-Associated Mitochondrial Dysfunction (SAMD) (Passos et al., 2010). Despite the close interdependent relationship between senescence and SAMD, the true complexity of these interactions and their role in aging remains to be elucidated. For example, it is currently unclear how much of the mitochondrial dysfunction that has been observed at tissue level during aging is actually associated with senescence at a cellular level. Furthermore, despite its central contribution to the senescent phenotype (Correia-Melo et al., 2016), it is not clear how mitochondria become dysfunctional in senescence. Importantly, an understanding of the potential consequences of SAMD in the context of tissue aging is only beginning to emerge. In this review, we will explore the following hypotheses:

1. Senescence results in dysregulated mitophagy that drives SAMD; and
2. SAMD is a significant cause of (accelerated) aging.

We conclude that mitophagy, SAMD and SASP are tightly interlinked in cell senescence by a network of inter-related feedback signalling pathways and that SAMD may be an essential cause of metabolic dysfunction in aging.

## 2 Dysfunctional mitochondria accumulate in senescent cells

It is well established that not only cell size but also mitochondrial mass increases significantly in senescent cells (Table 1). Kinetic studies in stress-induced senescence showed that the increase in mitochondrial mass is a fast but not immediate process, occurring with a delay of 2–3 days after the peak in DNA damage but before a robust SASP is established (Passos et al., 2010). As with most other senescence phenotypes, mitochondrial accumulation has preferentially been studied in fibroblasts, but occurs also in senescent epithelial cells (Hara et al., 2013), hepatocytes (Correia-Melo et al., 2016), enterocytes (Jurk et al., 2014) or neurons that develop a senescence-like phenotype in response to persistent DNA damage (Jurk et al., 2012). In oncogene-induced senescence, the activity of the mitochondrial ‘gatekeeper’ protein pyruvate dehydrogenase is increased by simultaneous suppression of the PDH-inhibitory enzyme pyruvate dehydrogenase kinase 1 (PDK1) and induction of the PDH-activating enzyme pyruvate dehydrogenase phosphatase 2 (PDP2). The resulting combined activation of PDH enhanced the use of pyruvate in the tricarboxylic acid cycle, causing increased respiration and redox stress (Kaplon et al., 2013). In senescent cells, the expression of fission mediators Drp1 and Fis1 (Mai et al., 2010) and frequencies of both fusion and fission events (Dalle Pezze et al., 2014) are reduced, resulting in enhanced connectivity of the mitochondrial network.

**Table 1:** Metabolic changes in senescence and aging.

	Mitochondrial mass	MPP or respiratory coupling	ROS production
RS	Increased (Passos et al., 2007)	Decreased (Passos et al., 2010, Passos et al., 2007)	Increased (Passos et al., 2007)
OIS	Increased (Moiseeva et al., 2009)	Decreased (Moiseeva et al., 2009)	Increased (Moiseeva et al., 2009)
SIS	Increased (Passos et al., 2010, Tai et al., 2017)	Decreased (Passos et al., 2010, Passos et al., 2007)	Increased (Passos et al., 2010, Tai et al., 2017)
Aging in vivo	Different results depending on tissue and methodology	Decreased e.g. liver (Miwa et al., 2014); brain (Cocco et al., 2009); skeletal muscle (Porter et al., 2015)	Increased e.g. liver (Miwa et al., 2014); heart (Petrosillo et al., 2009); skeletal muscle (Mansouri et al., 2006)

RS, Replicative senescence; OIS, Oncogene-induced senescence; SIS, Stress-induced senescence.

In functional mitochondria, oxygen uptake, ATP production, membrane potential and generation of ROS are tightly regulated to maintain the redox balance (Brand, 2016). While there is no simple correlation between membrane potential and superoxide production by the electron transport chain, mitochondria that accumulate in senescence often show a decreased membrane potential and at the same time produce increased levels of ROS (Table 1), suggesting dysfunctionality. In accordance with this notion, the capacity of senescent cells to regulate [Ca^2 +^]_i_ is decreased and a retrograde response is initiated (Passos et al., 2007). In mitochondria from senescent cells, the Respiratory Control Ratio (RCR, the ratio of oxygen uptake in state 3 (presence of ATP) to state 4 (presence of oligomycin)), is much lower than in young cells (Table 1), specifically if respiration is fuelled by complex I-linked substrates. Together, these data show that the mitochondria that accumulate during cell senescence are dysfunctional. We propose the notion of Senescence-Associated Mitochondrial Dysfunction (SAMD) for this phenotype.

**Fig. 1:**
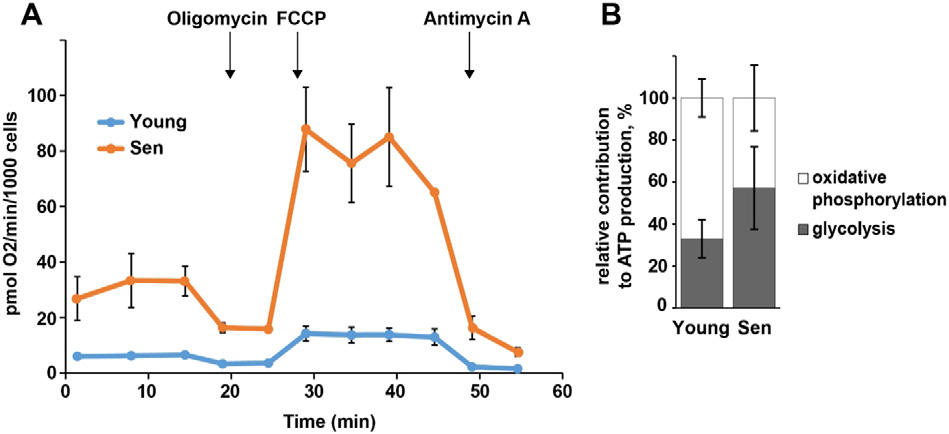
Senescence drives changes in cell bioenergetics. Human MRC5 fibroblasts were either fully proliferation competent (young) or replicatively senescent (sen). Cellular oxygen consumption rates and extracellular acidification rates were assayed in medium containing 5 mM glucose, 2 mM l-glutamate and 3% FBS using a Seahorse XF24 analyzer. A) Oxygen consumption rates showing basal condition, and after sequential additions of the following drugs as indicated: oligomycin (to block ATP synthesis), the uncoupler FCCP (to stimulate respiration) and Antimycin A (to block the electron transport chain at complex III). B) Relative glycolytic and mitochondrial ATP production rates were calculated using the data generated in Fig. 1A as described (Mookerjee and Brand, 2015). Data are mean ± SD from 20 samples from 2 independent experiments.

It should be noted that decreased activity of complex I, decreased coupling and increased ROS production accompany the aging process in many tissues (Table 1), paralleling the increase in the frequency of senescent cells in the same tissues (Jurk et al., 2014, Wang et al., 2009). Mitochondrial dysfunction has a profound effect on cellular bioenergetics (Fig. 1): First, increased mitochondrial mass is reflected by a significantly higher absolute oxygen consumption rate per cell in senescence (Fig. 1A). Second, in senescent cells the fraction of ATP produced by mitochondrial oxidative phosphorylation decreases, while relatively more ATP is generated by glycolysis (Fig. 1B). Thus, both the increase in mitochondrial abundance and the shift towards a more glycolytic mode of ATP production appear as compensatory responses to mitochondrial dysfunction. Evidently, early occurrence of mitochondrial dysfunction during the induction of cell senescence could set off a number of different cellular responses and signalling pathways as well as reducing the capacity to respond to peak energy demands.

In ionizing radiation (IR)-induced senescence, activation of p53 causes induction of the master regulator of mitochondrial biogenesis, peroxisome proliferator-activated receptor gamma, coactivator 1 beta (PGC-1β) driving an increase in mitochondrial mass. This is not due to a direct transactivation of PGC-1β, rather, p53 triggers murine double minute 2 (MDM2)-mediated hypoxia inducible factor 1 α (HIF-1α) degradation, leading to release of PGC-1β inhibition by HIF-1α (Bartoletti-Stella et al., 2013). This is in accordance with data showing HIF-1a as inducer of mitophagy (Allen et al., 2013). In apparent contrast, Sahin et al. (2011) described profound repression of both PGC-1α and PGC-1β, associated with impaired mitochondrial biogenesis, in mice null for either telomerase reverse transcriptase (*TERT*) or telomerase RNA component (*TERC*) genes. Telomerase knock-out mice show enhanced frequencies of senescent cells (Choudhury et al., 2007, Jurk et al., 2012, Jurk et al., 2014, Passos et al., 2010) and mitochondrial dysfunction (Spilsbury et al., 2015) in multiple tissues. A kinetic quantitative analysis of the induction of stress-dependent senescence resolved this contradiction (Dalle Pezze et al., 2014): PGC-1α (and presumably PGC-1β) was upregulated only temporarily during the early DNA damage response phase, but became down-regulated in established senescence. This transient upregulation caused only a minor increase in mitochondrial mass, while the majority of mitochondrial accumulation occurred later, when PGC-1β was already downregulated below control values. With progression of senescence, mitochondria accumulated into a progressively more rigid network characterized by low fusion and fission rates. Together with functional deterioration of accumulating mitochondria this suggested that a delay in autophagic degradation of defective mitochondria via a process called mitophagy (see next chapter) is a major contributor to mitochondria accumulation in stress-induced senescence.

## 3 Autophagy is an essential mitochondrial quality control mechanism implicated in senescence

### 3.1 Autophagy and cell senescence

**Fig. 2:**
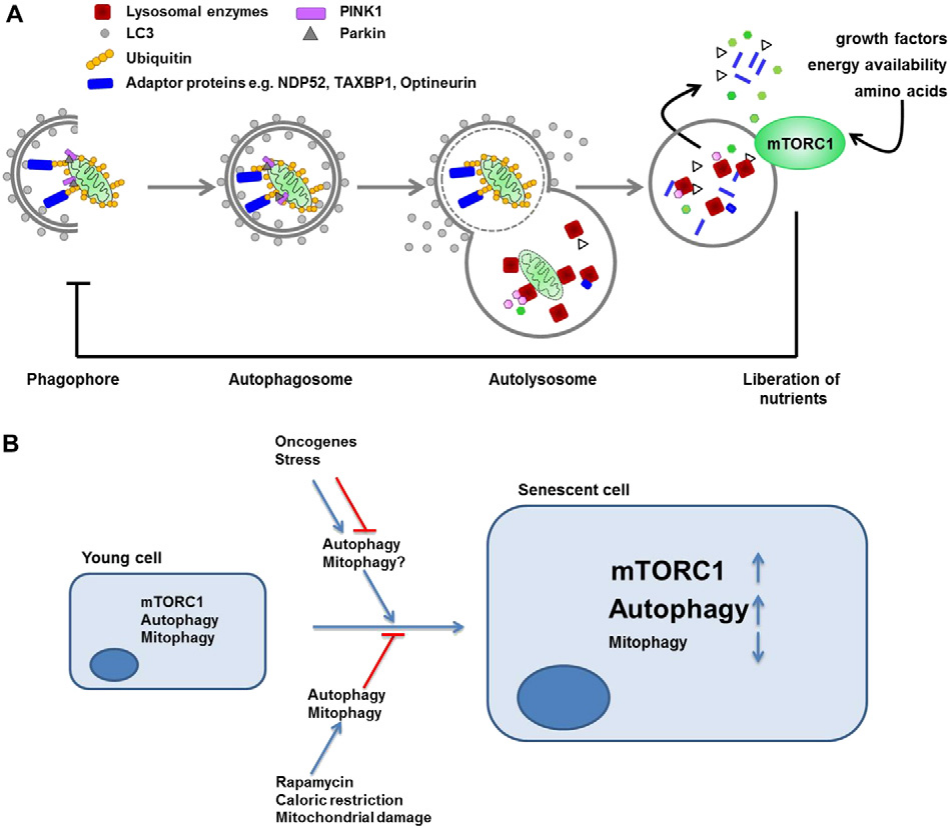
The potential role of mitophagy in senescence. A) Diagram showing the process of autophagy, exemplified by mitophagy. Cytoplasmic contents including organelles such as mitochondria are sequestered into double-membraned phagophore structures. This process can be selective and involves a number of receptor proteins; specifically for mitochondria these include NDP52, optineurin and TAXBP1. These receptor proteins bind to poly-ubiquitinated proteins which, via binding to the phagophore-resident protein LC3, recruit their cargo to the growing autophagosome. Ubiquitination of mitochondrial surface proteins can occur via stabilisation of PINK1 (PTEN-induced kinase 1) on the mitochondrial surface, which recruits the E3 ubiquitin ligase, Parkin to mitochondrial membranes, thus promoting the ubiquitination and clearance of damaged mitochondria. The fully enclosed autophagosome is trafficked to and fuses with lysosomes. The contents of the subsequent autolysosome are degraded by lysosomal enzymes including hydrolases and proteases. mTORC1 integrates intra- and extra-cellular signals and drives cell growth as well as inhibiting autophagy. B) In young proliferating cells, mTORC1 carefully balances anabolic vs catabolic metabolism in response to the cellular environment. Both mTORC1 activity and autophagy are elevated in senescent cells while the specific process of mitophagy is reduced (see text for more details). Autophagy has a complex relationship with senescence and can both promote and inhibit senescence and senescence-associated phenotypes. For example in the context of OIS, autophagy induction can either promote or suppress acquisition of senescence while the specific role of mitophagy in OIS is currently less clear. Conversely, lifespan-extending interventions such as caloric restriction and rapamycin induce autophagy and mitophagy and relieve senescence-associated phenotypes.

Autophagy is a collective term for several cellular pathways facilitating degradation of intracellular components by lysosomal proteases. Amongst autophagy pathways macroautophagy (hereafter for simplicity called autophagy) is the best studied process (Fig. 2A). Autophagy begins with the engulfment of surplus or damaged proteins and entire organelles by double membraned vesicles called autophagosomes which eventually fuse with lysosomes. The resulting hybrid organelle is called autolysosome and allows degradation of autophagy substrates and subsequent release of basic components such amino acids, lipids and nucleotides back into the cytoplasm for biosynthetic processes and energy generation. Autophagy activity is increased in conditions of nutrient deprivation sensed via the mechanistic Target of Rapamycin Complex 1 (mTORC1) pathway and serves as a recycling process necessary for cell survival during starvation. Importantly, autophagy is also a quality control mechanism selectively scavenging potentially toxic cellular components. Selective autophagy is classified on the basis of specific substrates with mitophagy being the most relevant to the topic of this review. Autophagy has been implicated in the development of cellular senescence. However, this relationship appears to be complex and often contradictory (Fig. 2B). Thus, on one hand autophagy has been suggested to act as a pro-senescence mechanism. Indeed, studies have demonstrated that autophagy promotes the process of oncogene-induced senescence (OIS). Overexpression of the autophagy gene ULK3 was found to enhance autophagy and stimulate senescence induced by the RAS oncogene in human fibroblasts (Young et al., 2009). This effect of autophagy was suggested to be mediated by a specialized intracellular structure called TOR-autophagy spatial coupling compartment (TASCC) (Narita et al., 2011). TASCC was shown to allow simultaneous and compartmentalised generation of amino acids and other metabolites by autophagy. Conversely, activation of the mTORC1 signalling pathway, a negative regulator of TASCC, promoted increased production of SASP factors thus facilitating acquisition of senescence phenotypes. Knockdown of the essential autophagy genes *Atg5* and *Atg7* was found to decrease SASP and bypass senescence (Narita et al., 2011, Young et al., 2009). Likewise, overexpression of the inhibitors of cyclin-dependent kinases p16(INK4A), p19(ARF) or p21(WAF1/CIP1) induced not only senescence as expected but also autophagy (Capparelli et al., 2012). Furthermore, autophagy induction by a proteolytic Cyclin E fragment (p18-CycE) was shown to facilitate DNA-damage-induced senescence (Singh et al., 2012).

At the same time, autophagy is well accepted as a protective, anti-aging process (Rubinsztein et al., 2011) and, consistent with this notion, a number of reports strongly implicated autophagy in the prevention of cellular senescence. In young, proliferating cells, inhibition of autophagy leads to senescence, at least in part mediated by accumulation of reactive oxygen species and activation of the tumour suppressor and senescence inducer protein p53, suggesting that autophagy suppresses senescence (Kang et al., 2011). Similarly, in models of mitochondria-targeted damage or oxidative-stress induced senescence, autophagy was shown to retard acquisition of senescence. Mitochondrial damage increased expression of autophagy genes *LC3B*, *ATG5* and *ATG12* and enhanced mitophagy which postponed senescence and enhanced replicative lifespan while peroxide treatment impaired autophagy flux resulting in senescence (Mai et al., 2012, Tai et al., 2017). Activation of autophagy by inhibition of mTORC1 was shown to efficiently suppress senescence phenotypes in a number of studies (Demidenko and Blagosklonny, 2008, Correia-Melo et al., 2016, Tai et al., 2017). In contrast to the earlier reports suggesting an OIS-promoting role of autophagy (Narita et al., 2011, Young et al., 2009) more recent reports have shown that autophagy can act as a mechanism to bypass RAS-induced senescence and facilitate tumour growth (Wang et al., 2012).

This contradictory role of autophagy in senescence may be explained, at least in part, by the complex nature of the autophagy process. For example, increased numbers of autophagic vesicles could indicate either increased autophagy or reduced flux, e.g. due to defective trafficking, fusion with lysosomes or defective lysosomal compartment. Moreover, different levels of autophagic activity can mediate different cellular responses to stressors, as it has been suggested in case of RAS-induced senescence. Thus, high levels of autophagy may prevent OIS acquisition, low levels may accompany it whilst more stringent inhibition of autophagy may promote senescence (Wang et al., 2012). Moreover, timing of autophagy inhibition is likely to be an essential factor for the development of senescence. As described above, rendering autophagy dysfunctional in young cells leads to senescence acquisition whilst in senescent cells autophagy frequently appears to become upregulated, which may be important for the establishment of senescence phenotypes. Finally, a number of senescence regulators that either activate or suppress senescence can be degraded by autophagy. Therefore, inhibition of autophagy may differentially impact on the process of senescence acquisition depending on the relative expression of these autophagy substrates, type of inhibition etc. As an example, it has been suggested that selective autophagy may suppress senescence through the degradation of the pro-senescence factor GATA4 whilst senescence may indeed be promoted by general autophagy through the TASCC (Kang et al., 2015, Narita et al., 2011).

### 3.2 Mitophagy and cell senescence

Similar to other selective autophagy pathways, in mitophagy, specificity is accomplished by a number of dedicated proteins, specifically the PTEN-induced putative kinase PINK1 and Parkin, which ensure that mitochondria with reduced membrane potential are targeted to nascent autophagic vesicles by tagging them with polyubiquitin chains (Fig. 2A). Ubiquitylated mitochondria are recognized by a set of receptor proteins including optineurin, NDP52 and TaxBP1 which in turn recruit autophagic membranes engulfing and isolating mitochondria. Mitophagy is important for the maintenance of cellular function and viability as evidenced from familial age-related diseases caused by mutations in genes encoding mitophagy components (Deas et al., 2011).

Interestingly, the regulation and functional role of mitophagy in cell senescence appears to be at least in part independent of changes in general autophagy (Fig. 2B). While general autophagy may become elevated or reduced depending on the model of senescence, mitophagy activity is reduced in senescent cells in vitro and in vivo (Dalle Pezze et al., 2014, Garcia-Prat et al., 2016). Several potential mechanisms may be responsible for this impairment of mitophagy activity. It may be a consequence of reduced basal or induced autophagic activity, which in turn may result from changes in its upstream regulators like mTOR. Indeed, in senescent cells activity of mTORC1 appears to be elevated (Correia-Melo et al., 2016, Dalle Pezze et al., 2014, Demidenko and Blagosklonny, 2008) and become unresponsive to starvation signals. Likewise, lysosomal overload will disrupt the terminal events of substrate degradation in both general autophagy and mitophagy pathways (Evangelou et al., 2017). Indeed, despite an extensive expansion of the lysosomal compartment during cell senescence, lysosomal activity is reduced and lysosomes accumulate undegraded material called lipofuscin not only during aging but also in senescence (Sitte et al., 2001). Lysosomal dysfunction may, at least in part, be driven by growth arrest.

Alternatively, mitophagy may become impaired due to mechanisms independent of general autophagy. Cytoplasmic p53, which accumulates in senescence, interacts with Parkin and prevents its translocation to dysfunctional mitochondria, thus suppressing mitophagy and stabilising senescence (Ahmad et al., 2015). Similarly, expression of PINK1 is low in aging lungs, suppressing mitophagy and enhancing the probability of senescence transition (Bueno et al., 2015). PINK1 deficiency also suppresses the process of mitochondrial fission, which is important for mitophagy by facilitating the separation of dysfunctional portions from the mitochondrial network. Reduced dynamics and increased fusion of the mitochondrial network in senescent cells has been proposed to result in the failure to sequester defective components of mitochondrial network for degradation via mitophagy (Dalle Pezze et al., 2014). As a result, “old” mitochondria avoid degradation while newly synthesised isolated mitochondria are turned over by mitophagy.

## 4 SAMD as a cause of senescence and aging

Data reviewed so far suggest that senescence is associated with dysregulated mitophagy and a subsequent accumulation of dysfunctional mitochondria. This senescence-associated mitochondrial dysfunction is a significant trigger of multiple dimensions of the senescent phenotype. Dysfunctional mitochondria in senescent cells produce enhanced levels of ROS which aggravate DNA damage and the DNA damage response signalling pathway (DDR), contributing to the development and stability of the senescent growth arrest (Passos et al., 2010). Moreover, dysfunctional mitochondria release multiple forms of “intracellular danger-associated molecular patterns” (DAMPs), especially ROS and mtDNA fragments, that are recognized by NOD-like receptors (NLR), especially NLRP3, triggering inflammasome assembly and activation of pro-inflammatory cytokines IL-1β and IL-18 (Salminen et al., 2012). Enhanced autophagic clearance of mitochondria suppressed NLRP3 activation (Nakahira et al., 2011, Zhou et al., 2011). This pathway interacts positively with NF-κB signalling enhancing transcription of many pro-inflammatory genes. Thus, mitophagy failure leading to SAMD may be responsible for the pro-inflammatory arm of the SASP. Interestingly, mitochondrial dysfunction (induced by depletion of mtDNA, mitochondrial sirtuins or by inhibition of the mitochondrial electron transport chain) that did not cause elevated cytoplasmic ROS and did not activate the DDR induced a senescent state that lacked the expression of the vast majority of the cytokines usually seem as part of the SASP (Wiley et al., 2016). Moreover, specific removal of all mitochondria in senescing human fibroblasts by activation of the ubiquitin ligase Parkin rescued the hyperproduction of ROS together with all other examined markers of the senescent phenotype (enlarged cell size, sen-β-Gal production, CDKN1A (p21) and CDKN2A (p16) expression, SASP) except growth arrest (Correia-Melo et al., 2016). The same suppression of the senescent phenotype including major SASP components has been achieved by treatment of cells or mice with the mTOR inhibitor and autophagy activator, rapamycin (Demidenko and Blagosklonny, 2008, Correia-Melo et al., 2016). Together, these data strongly implicate SAMD as a major driver of the senescent phenotype, especially of the SASP.

**Fig. 3:**
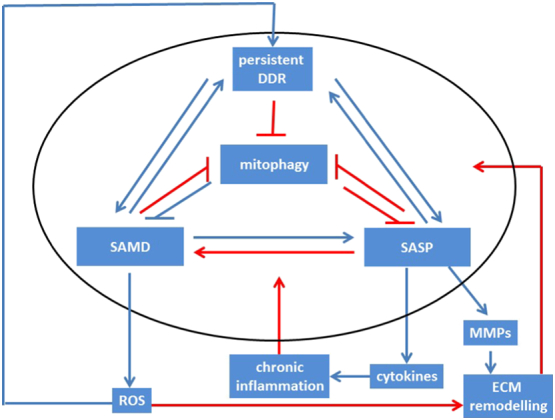
The cell senescence signalling network. Signals downstream of the DDR cause mitochondrial dysfunction via p38MAPK and TGFβ, SASP and chronic inflammation via NF-κB and C/EBPβ and may directly or indirectly suppress autophagy and mitophagy. SASP and mitochondrial dysfunction feedback to maintain and enhance DNA damage at least partly via ROS generation. Many potential interactions within the network (indicated by red arrows) are still insufficiently understood. See text for further details.

Importantly, the interconnections between SAMD, SASP and cell senescence do not form a simple linear cause-effect relationship. There is ample evidence for the existence of multiple positive feedback loops between the major components of the senescent phenotype (Fig. 3). Positive feedback loops between SASP factors and DDR (Acosta et al., 2008, Kuilman et al., 2008) or between SAMD and DDR (Passos et al., 2010) are well documented. It has also been shown that chronic activation of NF-κB not only enhances the SASP but also aggravates ROS production and the DDR in senescence (Jurk et al., 2014) possibly at least in part via a ‘non-canonical’ function of NLRP3. There is also evidence for intriguing interactions between SASP, SAMD and the extracellular matrix (ECM) (Fig. 3): Senescent cells are known to negatively impact their surrounding environment, including both bystander cells and composition and structure of the ECM via secretion of matrix metalloproteinases, especially MMP-1, and decreased expression of its endogenous inhibitor TIMP-1 as part of the SASP, resulting in ECM degradation with aging (Brennan et al., 2003). SAMD may play an important role for these degenerative changes, as they can be triggered by retrograde signalling in response to mtDNA mutations and electron transport chain dysfunction (Krutmann and Schroeder, 2009, Wiley et al., 2016). ECM collagenolysis autonomously inhibits further pro-collagen synthesis and down-regulates hyaluronic acid synthase 2 (Rock et al., 2011), further contributing to ECM degeneration. Hyaluronic acid is a linear polysaccharide with pro- or anti-proliferative properties depending on its molecular weight; the extremely high molecular weight hyaluronic acid produced by naked mole rats is a major determinant of their remarkable tumour resistance because of its strong anti-proliferative effect (Tian et al., 2013). It is thus probable that ECM remodelling by senescent cells feeds back into aggravation of the senescent phenotype and mitochondrial dysfunction in the matrix-embedded cells. Together, these data show that mitochondrial dysfunction is both a cause and a consequence of senescence. Thus, mitochondrial turnover and especially mitophagy might be the central regulator of cell senescence (Fig. 3).

## 5 Outstanding questions

Mitochondrial dysfunction is well accepted as a driver of tissue and organism aging with potential to modulate aging either positively or negatively depending on a set of insufficiently understood conditions (Wang and Hekimi, 2015). An important question is to what extent aging-associated mitochondrial dysfunction and cell senescence/SAMD are interrelated. Does aging-related mitochondrial dysfunction cause senescence in vivo or vice versa? Is mitochondrial dysfunction in aging actually a mosaic phenomenon, occurring preferentially or exclusively in the senescent cells? Given the high prevalence of senescent cells in many tissues (see above), this appears highly possible. Emerging data suggest that it is SAMD rather more than general loss of mitochondrial function in aging that reduces homeostatic capability, causing compromised responses to peak energy demand and driving metabolic insufficiency in aging. For instance, we have found that SAMD in hepatocytes (and other cell types) includes a compromised capacity to metabolize fatty acids, which causes cell-autonomous lipid storage in aging liver and thus contributes to fatty liver (steatosis), a common and pathologically significant complication of liver aging (Ogrodnik et al. unpublished). Adipocyte senescence is an essential driver of adipose tissue dysfunction and obesity (Stout et al., 2017), and this link is very probably mediated by SAMD. Analysing mitochondrial dysfunction in aging tissues at single-cell resolution in combination with interventions that selectively ablate senescent cells (senolytics) will enable a better understanding of the importance of SAMD in aging.

SAMD appears to be a central mediator for interventions that extend healthy lifespan. Dietary restriction induces autophagy, improves mitochondrial function, specifically in complex I (Miwa et al., 2014) and reduces frequencies of senescent cells in many tissues (Wang et al., 2010). Similarly the dietary restriction mimetic rapamycin, which also prolongs healthy lifespan in many species by activating autophagy (Kennedy and Lamming, 2016) and improving mitochondrial complex I function (Miwa et al., 2014), acts as a senostatic, i.e. it inhibits multiple aspects of the senescent phenotype including SAMD (Demidenko and Blagosklonny, 2008, Correia-Melo et al., 2016). Recently, metformin gained increasing attention as a drug that can enhance lifespan and healthspan not only in rodents but also in man (Bannister et al., 2014). Metformin improves mitochondrial complex I function (Foretz et al., 2014), inhibits mTORC1 signalling by activating AMPK (Howell et al., 2016) and blocks pro-inflammatory NF-κB signalling (Saisho, 2015). While metformin may sensitize some cell types to stress-induced senescence in vitro, it inhibits the SASP, thus blocking senescent cell-induced bystander effects (Moiseeva et al., 2013) and may act as a senostatic drug in vivo similar to rapamycin.

Senescent cells typically upregulate anti-apoptotic pathways, and are preferentially susceptible to inhibition of these pro-survival mechanisms. This has been dubbed the ‘Achilles heel’ of senescent cells and used as a rationale for the development of senostatic drugs (Kirkland & Tchkonia, in this volume). It is tempting to speculate that the low mitochondrial membrane potential found in many senescent cells lies at the heart of this preferential sensitivity by easing the release of apoptosis-stimulating factors from mitochondria. Interestingly in this respect, a recent screen of compounds that modify autophagy (and possibly mitophagy) identified a large number of these as potential senolytics (Fuhrmann-Stroissig et al., unpublished). It seems probable that for drugs to effectively extend such a multifaceted phenotype as healthy lifespan they need to target not just a singular molecular node but a complex network of aging-determining pathways and mechanisms like the one defined by the interactions between mitophagy, SAMD and SASP in cell senescence (see Fig. 3). We believe that improved understanding of this network will offer an exciting avenue in the development of novel interventions that can prolong healthy lifespan including senolytic and senostatic agents.

## Author contributions

VIK and TvZ designed the review. SM and BC contributed individual parts and data. TvZ wrote the review with input from all authors.

## Conflict of interests

None.

## References

[bb0005] Acosta J.C., O'loghlen A., Banito A., Guijarro M.V., Augert A., Raguz S., Fumagalli M., Da Costa M., Brown C., Popov N., Takatsu Y., Melamed J., D'adda Di Fagagna F., Bernard D., Hernando E., Gil J. (2008). Chemokine signaling via the CXCR2 receptor reinforces senescence. Cell.

[bb0010] Ahmad T., Sundar I.K., Lerner C.A., Gerloff J., Tormos A.M., Yao H., Rahman I. (2015). Impaired mitophagy leads to cigarette smoke stress-induced cellular senescence: implications for chronic obstructive pulmonary disease. FASEB J..

[bb0015] Akbar A.N., Henson S.M., Lanna A. (2016). Senescence of T lymphocytes: implications for enhancing human immunity. Trends Immunol..

[bb0020] Allen G.F., Toth R., James J., Ganley I.G. (2013). Loss of iron triggers PINK1/Parkin-independent mitophagy. EMBO Rep..

[bb0025] Baker D.J., Childs B.G., Durik M., Wijers M.E., Sieben C.J., Zhong J., Saltness R.A., Jeganathan K.B., Verzosa G.C., Pezeshki A., Khazaie K., Miller J.D., Van Deursen J.M. (2016). Naturally occurring p16(Ink4a)-positive cells shorten healthy lifespan. Nature.

[bb0030] Bannister C.A., Holden S.E., Jenkins-Jones S., Morgan C.L., Halcox J.P., Schernthaner G., Mukherjee J., Currie C.J. (2014). Can people with type 2 diabetes live longer than those without? A comparison of mortality in people initiated with metformin or sulphonylurea monotherapy and matched, non-diabetic controls. Diabetes Obes. Metab..

[bb0035] Bartoletti-Stella A., Mariani E., Kurelac I., Maresca A., Caratozzolo M.F., Iommarini L., Carelli V., Eusebi L.H., Guido A., Cenacchi G., Fuccio L., Rugolo M., Tullo A., Porcelli A.M., Gasparre G. (2013). Gamma rays induce a p53-independent mitochondrial biogenesis that is counter-regulated by HIF1alpha. Cell Death Dis..

[bb0040] Brand M.D. (2016). Mitochondrial generation of superoxide and hydrogen peroxide as the source of mitochondrial redox signaling. Free Radic. Biol. Med..

[bb0045] Brennan M., Bhatti H., Nerusu K.C., Bhagavathula N., Kang S., Fisher G.J., Varani J., Voorhees J.J. (2003). Matrix metalloproteinase-1 is the major collagenolytic enzyme responsible for collagen damage in UV-irradiated human skin. Photochem. Photobiol..

[bb0050] Bueno M., Lai Y.C., Romero Y., Brands J., St Croix C.M., Kamga C., Corey C., Herazo-Maya J.D., Sembrat J., Lee J.S., Duncan S.R., Rojas M., Shiva S., Chu C.T., Mora A.L. (2015). PINK1 deficiency impairs mitochondrial homeostasis and promotes lung fibrosis. J. Clin. Invest..

[bb0055] Capparelli C., Chiavarina B., Whitaker-Menezes D., Pestell T.G., Pestell R.G., Hulit J., Ando S., Howell A., Martinez-Outschoorn U.E., Sotgia F., Lisanti M.P. (2012). CDK inhibitors (p16/p19/p21) induce senescence and autophagy in cancer-associated fibroblasts, "fueling" tumor growth via paracrine interactions, without an increase in neo-angiogenesis. Cell Cycle.

[bb0060] Choudhury A.R., Ju Z., Djojosubroto M.W., Schienke A., Lechel A., Schaetzlein S., Jiang H., Stepczynska A., Wang C., Buer J., Lee H.W., Von Zglinicki T., Ganser A., Schirmacher P., Nakauchi H., Rudolph K.L. (2007). Cdkn1a deletion improves stem cell function and lifespan of mice with dysfunctional telomeres without accelerating cancer formation. Nat. Genet..

[bb0065] Cocco T., Pacelli C., Sgobbo P., Villani G. (2009). Control of OXPHOS efficiency by complex I in brain mitochondria. Neurobiol. Aging.

[bb0070] Coppe J.P., Patil C.K., Rodier F., Sun Y., Munoz D.P., Goldstein J., Nelson P.S., Desprez P.Y., Campisi J. (2008). Senescence-associated secretory phenotypes reveal cell-nonautonomous functions of oncogenic RAS and the p53 tumor suppressor. PLoS Biol..

[bb0075] Correia-Melo C., Marques F.D., Anderson R., Hewitt G., Hewitt R., Cole J., Carroll B.M., Miwa S., Birch J., Merz A., Rushton M.D., Charles M., Jurk D., Tait S.W., Czapiewski R., Greaves L., Nelson G., Bohlooly Y.M., Rodriguez-Cuenca S., Vidal-Puig A., Mann D., Saretzki G., Quarato G., Green D.R., Adams P.D., Von Zglinicki T., Korolchuk V.I., Passos J.F. (2016). Mitochondria are required for pro-ageing features of the senescent phenotype. EMBO J..

[bb0080] Dalle Pezze P., Nelson G., Otten E.G., Korolchuk V.I., Kirkwood T.B., Von Zglinicki T., Shanley D.P. (2014). Dynamic modelling of pathways to cellular senescence reveals strategies for targeted interventions. PLoS Comput. Biol..

[bb0085] Deas E., Wood N.W., Plun-Favreau H. (2011). Mitophagy and Parkinson's disease: the PINK1-parkin link. Biochim. Biophys. Acta.

[bb0090] Demaria M., O'leary M.N., Chang J., Shao L., Liu S., Alimirah F., Koenig K., Le C., Mitin N., Deal A.M., Alston S., Academia E.C., Kilmarx S., Valdovinos A., Wang B., De Bruin A., Kennedy B.K., Melov S., Zhou D., Sharpless N.E., Muss H., Campisi J. (2017). Cellular senescence promotes adverse effects of chemotherapy and cancer relapse. Cancer Discov..

[bb0095] Demidenko Z.N., Blagosklonny M.V. (2008). Growth stimulation leads to cellular senescence when the cell cycle is blocked. Cell Cycle.

[bb0100] Dimri G.P., Lee X., Basile G., Acosta M., Scott G., Roskelley C., Medrano E.E., Linskens M., Rubelj I., Pereira-Smith O. (1995). A biomarker that identifies senescent human cells in culture and in aging skin in vivo. Proc. Natl. Acad. Sci. U. S. A..

[bb0105] Evangelou K., Lougiakis N., Rizou S., Kotsinas K., Kletsas D., Muñoz-Espín D., Kastrinakis N.G., Pouli N., Marakos P., Townsend P., Serrano M., Bartek J., Gorgoulis V.G. (2017). Robust, universal biomarker assay to detect senescent cells in biological specimens. Aging Cell.

[bb0110] Farr J.N., Fraser D.G., Wang H., Jaehn K., Ogrodnik M.B., Weivoda M.M., Drake M.T., Tchkonia T., Lebrasseur N.K., Kirkland J.L., Bonewald L.F., Pignolo R.J., Monroe D.G., Khosla S. (2016). Identification of senescent cells in the bone microenvironment. J. Bone Miner. Res..

[bb0115] Finkel T. (2015). The metabolic regulation of aging. Nat. Med..

[bb0120] Foretz M., Guigas B., Bertrand L., Pollak M., Viollet B. (2014). Metformin: from mechanisms of action to therapies. Cell Metab..

[bb0125] Garcia-Prat L., Martinez-Vicente M., Perdiguero E., Ortet L., Rodriguez-Ubreva J., Rebollo E., Ruiz-Bonilla V., Gutarra S., Ballestar E., Serrano A.L., Sandri M., Munoz-Canoves P. (2016). Autophagy maintains stemness by preventing senescence. Nature.

[bb0130] Hara H., Araya J., Ito S., Kobayashi K., Takasaka N., Yoshii Y., Wakui H., Kojima J., Shimizu K., Numata T., Kawaishi M., Kamiya N., Odaka M., Morikawa T., Kaneko Y., Nakayama K., Kuwano K. (2013). Mitochondrial fragmentation in cigarette smoke-induced bronchial epithelial cell senescence. Am. J. Phys. Lung Cell. Mol. Phys..

[bb0135] Howell J.J., Hellberg K., Turner M., Talbott G., Kolar M.J., Ross D.S., Hoxhaj G., Saghatelian A., Shaw R.J., Manning B.D. (2016). Metformin inhibits hepatic mTORC1 signaling via dose-dependent mechanisms involving AMPK and the TSC complex. Cell Metab..

[bb0140] Jurk D., Wang C., Miwa S., Maddick M., Korolchuk V., Tsolou A., Gonos E.S., Thrasivoulou C., Saffrey M.J., Cameron K., Von Zglinicki T. (2012). Postmitotic neurons develop a p21-dependent senescence-like phenotype driven by a DNA damage response. Aging Cell.

[bb0145] Jurk D., Wilson C., Passos J.F., Oakley F., Correia-Melo C., Greaves L., Saretzki G., Fox C., Lawless C., Anderson R., Hewitt G., Pender S.L., Fullard N., Nelson G., Mann J., Van De Sluis B., Mann D.A., Von Zglinicki T. (2014). Chronic inflammation induces telomere dysfunction and accelerates ageing in mice. Nat. Commun..

[bb0150] Kang C., Xu Q., Martin T.D., Li M.Z., Demaria M., Aron L., Lu T., Yankner B.A., Campisi J., Elledge S.J. (2015). The DNA damage response induces inflammation and senescence by inhibiting autophagy of GATA4. Science.

[bb0155] Kang H.T., Lee K.B., Kim S.Y., Choi H.R., Park S.C. (2011). Autophagy impairment induces premature senescence in primary human fibroblasts. PLoS One.

[bb0160] Kaplon J., Zheng L., Meissl K., Chaneton B., Selivanov V.A., Mackay G., Van Der Burg S.H., Verdegaal E.M., Cascante M., Shlomi T., Gottlieb E., Peeper D.S. (2013). A key role for mitochondrial gatekeeper pyruvate dehydrogenase in oncogene-induced senescence. Nature.

[bb0165] Kennedy B.K., Lamming D.W. (2016). The mechanistic target of rapamycin: the grand ConducTOR of metabolism and aging. Cell Metab..

[bb0170] Krutmann J., Schroeder P. (2009). Role of mitochondria in photoaging of human skin: the defective powerhouse model. J. Investig. Dermatol. Symp. Proc..

[bb0175] Kuilman T., Michaloglou C., Vredeveld L.C., Douma S., Van Doorn R., Desmet C.J., Aarden L.A., Mooi W.J., Peeper D.S. (2008). Oncogene-induced senescence relayed by an interleukin-dependent inflammatory network. Cell.

[bb0180] Lopez-Otin C., Blasco M.A., Partridge L., Serrano M., Kroemer G. (2013). The hallmarks of aging. Cell.

[bb0185] Mai S., Klinkenberg M., Auburger G., Bereiter-Hahn J., Jendrach M. (2010). Decreased expression of Drp1 and Fis1 mediates mitochondrial elongation in senescent cells and enhances resistance to oxidative stress through PINK1. J. Cell Sci..

[bb0190] Mai S., Muster B., Bereiter-Hahn J., Jendrach M. (2012). Autophagy proteins LC3B, ATG5 and ATG12 participate in quality control after mitochondrial damage and influence lifespan. Autophagy.

[bb0195] Mansouri A., Muller F.L., Liu Y., Ng R., Faulkner J., Hamilton M., Richardson A., Huang T.T., Epstein C.J., Van Remmen H. (2006). Alterations in mitochondrial function, hydrogen peroxide release and oxidative damage in mouse hind-limb skeletal muscle during aging. Mech. Ageing Dev..

[bb0200] Miwa S., Jow H., Baty K., Johnson A., Czapiewski R., Saretzki G., Treumann A., Von Zglinicki T. (2014). Low abundance of the matrix arm of complex I in mitochondria predicts longevity in mice. Nat. Commun..

[bb0205] Moiseeva O., Bourdeau V., Roux A., Deschenes-Simard X., Ferbeyre G. (2009). Mitochondrial dysfunction contributes to oncogene-induced senescence. Mol. Cell. Biol..

[bb0210] Moiseeva O., Deschenes-Simard X., St-Germain E., Igelmann S., Huot G., Cadar A.E., Bourdeau V., Pollak M.N., Ferbeyre G. (2013). Metformin inhibits the senescence-associated secretory phenotype by interfering with IKK/NF-kappaB activation. Aging Cell.

[bb0215] Mookerjee S.A., Brand M.D. (2015). Measurement and Analysis of Extracellular Acid Production to Determine Glycolytic Rate. J. Vis. Exp..

[bb0220] Nakahira K., Haspel J.A., Rathinam V.A., Lee S.J., Dolinay T., Lam H.C., Englert J.A., Rabinovitch M., Cernadas M., Kim H.P., Fitzgerald K.A., Ryter S.W., Choi A.M. (2011). Autophagy proteins regulate innate immune responses by inhibiting the release of mitochondrial DNA mediated by the NALP3 inflammasome. Nat. Immunol..

[bb0225] Narita M., Young A.R., Arakawa S., Samarajiwa S.A., Nakashima T., Yoshida S., Hong S., Berry L.S., Reichelt S., Ferreira M., Tavare S., Inoki K., Shimizu S., Narita M. (2011). Spatial coupling of mTOR and autophagy augments secretory phenotypes. Science.

[bb0230] Nelson G., Wordsworth J., Wang C., Jurk D., Lawless C., Martin-Ruiz C., Von Zglinicki T. (2012). A senescent cell bystander effect: senescence-induced senescence. Aging Cell.

[bb0235] Passos J.F., Nelson G., Wang C., Richter T., Simillion C., Proctor C.J., Miwa S., Olijslagers S., Hallinan J., Wipat A., Saretzki G., Rudolph K.L., Kirkwood T.B., Von Zglinicki T. (2010). Feedback between p21 and reactive oxygen production is necessary for cell senescence. Mol. Syst. Biol..

[bb0240] Passos J.F., Saretzki G., Ahmed S., Nelson G., Richter T., Peters H., Wappler I., Birket M.J., Harold G., Schaeuble K., Birch-Machin M.A., Kirkwood T.B., Von Zglinicki T. (2007). Mitochondrial dysfunction accounts for the stochastic heterogeneity in telomere-dependent senescence. PLoS Biol..

[bb0245] Petrosillo G., Matera M., Moro N., Ruggiero F.M., Paradies G. (2009). Mitochondrial complex I dysfunction in rat heart with aging: critical role of reactive oxygen species and cardiolipin. Free Radic. Biol. Med..

[bb0250] Porter C., Hurren N.M., Cotter M.V., Bhattarai N., Reidy P.T., Dillon E.L., Durham W.J., Tuvdendorj D., Sheffield-Moore M., Volpi E., Sidossis L.S., Rasmussen B.B., Borsheim E. (2015). Mitochondrial respiratory capacity and coupling control decline with age in human skeletal muscle. Am. J. Physiol. Endocrinol. Metab..

[bb0255] Rock K., Grandoch M., Majora M., Krutmann J., Fischer J.W. (2011). Collagen fragments inhibit hyaluronan synthesis in skin fibroblasts in response to ultraviolet B (UVB): new insights into mechanisms of matrix remodeling. J. Biol. Chem..

[bb0260] Rubinsztein D.C., Marino G., Kroemer G. (2011). Autophagy and aging. Cell.

[bb0265] Sahin E., Colla S., Liesa M., Moslehi J., Muller F.L., Guo M., Cooper M., Kotton D., Fabian A.J., Walkey C., Maser R.S., Tonon G., Foerster F., Xiong R., Wang Y.A., Shukla S.A., Jaskelioff M., Martin E.S., Heffernan T.P., Protopopov A., Ivanova E., Mahoney J.E., Kost-Alimova M., Perry S.R., Bronson R., Liao R., Mulligan R., Shirihai O.S., Chin L., Depinho R.A. (2011). Telomere dysfunction induces metabolic and mitochondrial compromise. Nature.

[bb0270] Saisho Y. (2015). Metformin and inflammation: its potential beyond glucose-lowering effect. Endocr Metab Immune Disord Drug Targets.

[bb0275] Salminen A., Kaarniranta K., Kauppinen A. (2012). Inflammaging: disturbed interplay between autophagy and inflammasomes. Aging (Albany NY).

[bb0280] Schafer M.J., White T.A., Evans G., Tonne J.M., Verzosa G.C., Stout M.B., Mazula D.L., Palmer A.K., Baker D.J., Jensen M.D., Torbenson M.S., Miller J.D., Ikeda Y., Tchkonia T., Van Deursen J.M., Kirkland J.L., Lebrasseur N.K. (2016). Exercise prevents diet-induced cellular senescence in adipose tissue. Diabetes.

[bb0285] Singh K., Matsuyama S., Drazba J.A., Almasan A. (2012). Autophagy-dependent senescence in response to DNA damage and chronic apoptotic stress. Autophagy.

[bb0290] Sitte N., Merker K., Grune T., Von Zglinicki T. (2001). Lipofuscin accumulation in proliferating fibroblasts in vitro: an indicator of oxidative stress. Exp. Gerontol..

[bb0295] Spilsbury A., Miwa S., Attems J., Saretzki G. (2015). The role of telomerase protein TERT in Alzheimer's disease and in tau-related pathology in vitro. J. Neurosci..

[bb0300] Stout M.B., Justice J.N., Nicklas B.J., Kirkland J.L. (2017). Physiological aging: links among adipose tissue dysfunction, diabetes, and frailty. Physiology (Bethesda).

[bb0305] Tai H., Wang Z., Gong H., Han X., Zhou J., Wang X., Wei X., Ding Y., Huang N., Qin J., Zhang J., Wang S., Gao F., Chrzanowska-Lightowlers Z.M., Xiang R., Xiao H. (2017). Autophagy impairment with lysosomal and mitochondrial dysfunction is an important characteristic of oxidative stress-induced senescence. Autophagy.

[bb0310] Tian X., Azpurua J., Hine C., Vaidya A., Myakishev-Rempel M., Ablaeva J., Mao Z., Nevo E., Gorbunova V., Seluanov A. (2013). High-molecular-mass hyaluronan mediates the cancer resistance of the naked mole rat. Nature.

[bb0315] Wang C., Jurk D., Maddick M., Nelson G., Martin-Ruiz C., Von Zglinicki T. (2009). DNA damage response and cellular senescence in tissues of aging mice. Aging Cell.

[bb0320] Wang C., Maddick M., Miwa S., Jurk D., Czapiewski R., Saretzki G., Langie S.A., Godschalk R.W., Cameron K., Von Zglinicki T. (2010). Adult-onset, short-term dietary restriction reduces cell senescence in mice. Aging (Albany NY).

[bb0325] Wang Y., Hekimi S. (2015). Mitochondrial dysfunction and longevity in animals: untangling the knot. Science.

[bb0330] Wang Y., Wang X.D., Lapi E., Sullivan A., Jia W., He Y.W., Ratnayaka I., Zhong S., Goldin R.D., Goemans C.G., Tolkovsky A.M., Lu X. (2012). Autophagic activity dictates the cellular response to oncogenic RAS. Proc. Natl. Acad. Sci. U. S. A..

[bb0335] Wiley C.D., Velarde M.C., Lecot P., Liu S., Sarnoski E.A., Freund A., Shirakawa K., Lim H.W., Davis S.S., Ramanathan A., Gerencser A.A., Verdin E., Campisi J. (2016). Mitochondrial dysfunction induces senescence with a distinct secretory phenotype. Cell Metab..

[bb0340] Xu M., Palmer A.K., Ding H., Weivoda M.M., Pirtskhalava T., White T.A., Sepe A., Johnson K.O., Stout M.B., Giorgadze N., Jensen M.D., Lebrasseur N.K., Tchkonia T., Kirkland J.L. (2015). Targeting senescent cells enhances adipogenesis and metabolic function in old age. elife.

[bb0345] Young A.R., Narita M., Ferreira M., Kirschner K., Sadaie M., Darot J.F., Tavare S., Arakawa S., Shimizu S., Watt F.M., Narita M. (2009). Autophagy mediates the mitotic senescence transition. Genes Dev..

[bb0350] Zhou R., Yazdi A.S., Menu P., Tschopp J. (2011). A role for mitochondria in NLRP3 inflammasome activation. Nature.

